# Preoperative inflammation markers and IDH mutation status predict glioblastoma patient survival

**DOI:** 10.18632/oncotarget.15235

**Published:** 2017-02-09

**Authors:** Peng-Fei Wang, Hong-Wang Song, Hong-Qing Cai, Ling-Wei Kong, Kun Yao, Tao Jiang, Shou-Wei Li, Chang-Xiang Yan

**Affiliations:** ^1^ Department of Neurosurgery, Sanbo Brain Hospital, Capital Medical University, China; ^2^ Department of Neurosurgery, National Cancer Center/Cancer Hospital, Chinese Academy of Medical Sciences and Peking Union Medical College, China; ^3^ Department of Pathology, Sanbo Brain Hospital, Capital Medical University, China; ^4^ Department of Neurosurgery, Beijing Tiantan Hospital, Capital Medical University, Beijing, China; ^5^ Beijing Neurosurgical Institute, Beijing, China

**Keywords:** glioblastomas, IDH-1 R132H mutation, neutrophil to lymphocyte ratio, platelet to lymphocyte ratio, lymphocyte to monocyte ratio

## Abstract

Recent studies suggest that inflammation response biomarkers are prognostic indicators of solid tumor outcomes. Here, we quantify the prognostic value of the neutrophil-to-lymphocyte ratio (NLR), platelet-to-lymphocyte ratio (PLR), and lymphocyte-to-monocyte ratio (LMR) in glioblastomas (GBMs), taking into consideration the role of the isocitrate dehydrogenase (IDH) mutation status. We examined 141 primary glioblastomas (pGBMs) and 25 secondary glioblastomas (sGBMs). NLRs, PLRs, and LMRs were calculated before surgery. IDH mutations were detected immunohistochemically after tumor resection, and patients' clinical outcomes were analyzed after classification into GBM, pGBM, and IDH-wild type glioblastoma (IDH-wt GBM) groups. To make comparisons, we set cutoffs for NLR, PLR and LMR of 4.0, 175.0, and 3.7, respectively. In a multivariate analysis, both NLR (HR=1.712, 95% CI 1.026-2.858, p=0.040) and PLR (HR=2.051, 95% CI 1.288-3.267, p=0.002) had independent prognostic value. While a low NLR was associated with a better prognosis only in the IDH-wt GBM group, PLR was predictive of patient survival in the GBM, pGBM, and IDH-wt GBM groups. By contrast, LMR exhibited no prognostic value for any of the 3 types of GBM.

## INTRODUCTION

Glioblastomas are the most common brain malignancies, accounting for 15.1% of the total central nervous system tumors [1]. Glioblastomas are classified as either primary glioblastomas (pGBMs) or secondary glioblastomas (sGBMs), which develop from lower-grade gliomas [2]. The discovery that isocitrate dehydrogenase (IDH) mutations are more common in sGBMs was one of the most significant advancements in the understanding of gliomas [3, 4]. Patients with glioblastomas carrying IDH mutations or wildtype IDH, exhibited large differences in prognosis, age, and genetic alternations [5–9].

Mounting evidence suggests an important role for inflammation in tumor development [10, 11]. The development of gliomas, in particular, is closely associated with inflammation status and immune response [12, 13]. NLRs, PLRs, and LMRs are markers of host inflammation. A high NLR and PLR and low LMR are closely associated with a poor prognosis in solid malignancies, including gastrointestinal tumors, prostate cancer, and lung cancer [14–19]. While a low preoperative NLR closely correlates with lower glioma grade and better clinical outcome [20–22], there are no published data assessing the role of PLR or LMR in gliomas. Additionally, the role of the NLR in gliomas requires further study, due to the limited number of cases in previous studies [21, 22]. We therefore hypothesized that the inflammation status would likely vary according to the IDH mutation status, and could serve as a prognostic indicator. Herein, we investigated the prognostic value of NLRs, PLRs, and LMRs, in both pGBMs and sGBMs. The characteristics of NLRs, PLRs, and LMRs are also described here, taking into consideration IDH mutation status.

## RESULTS

### Patient characteristics

We enrolled 166 patients with GBMs in the present study, including 70 females and 96 males. The age of the patients ranged from 18 to 80 years with an average of 52.1 ± 0.984 years. The frequency of IDH mutations was 9.9% (14/141) among pGBMs and 68% (17/25) among sGBM. The Karnofsky score (KPS), tumor location, surgical resection, and molecular markers are described in Table 1.

**Table 1:** Characteristics of the study population

Variables	No.	mOS (95% CI) months	HR (95% CI)	*P*
Age				
< 60	110	14.70 (11.83-17.58)	1.48 (1.04-2.12)	0.032
≥60	56	9.63 (7.96-11.30)		
Gender				
female	70	12.27 (7.30-17.24)	1.16 (0.81-1.65)	0.419
male	96	12.80 (10.65-14.95)		
Preoperative KPS				
≤70	82	10.67 (7.94-13.40)	1.48 (1.04-2.09)	0.028
>70	84	16.17 (12.81-19.53)		
Pathology				
pGBM	141	13.00 (10.42-15.58)	1.37 (0.88-2.12)	0.164
sGBM	25	10.67 (5.82-15.52)		
Location				
Frontal	33	11.97 (8.86-15.08)	1.04 (0.93-1.15)	0.504
Temporal	27	17.00 (10.14-23.87)		
Parietal	12	10.96 (4.80-17.12)		
Other site	19	8.37 (3.02-13.72)		
Mixed	75	13.33 (9.45-17.21)		
Size				
≤ 5 cm	68	13.33 (7.86-18.80)	0.95 (0.67-1.35)	0.760
> 5 cm	98	12.27 (10.34-14.20)		
Resection				
GTR	102	13.33 (10.50-16.26)	1.47 (1.03-2.09)	0.033
non-GTR	64	9.40 (5.93-12.87)		
Standard treatment				
yes	114	14.87 (12.08-17.66)	2.42 (1.67-3.50)	0.000
no	52	7.9 (4.44-11.36)		
IDH-1R132H				
mutant	31	17.17 (8.84-25.50)	1.60 (1.01-2.52)	0.043
wild-type	135	12.00 (9.34-14.66)		

The median overall survival (OS) did not differ with respect to gender, tumor location, or tumor size (Table 1). Patients carrying IDH-1^R132H^ mutations had better prognoses [median 17.17 months (95% CI 8.84 – 25.50) vs. 12.00 months (95% CI 9.34 – 14.66); *p* = 0.041]. Higher preoperative KPS, surgical resection, and full treatment with radiochemotherapy were also associated with better clinical outcomes (Table 1). Among patients meeting our inclusion criteria, these clinical characteristics varied within a reasonable range in previous reports [1, 2, 5, 8].

### No association between NLR, PLR, or LMR and IDH mutations

We observed that NLR was elevated more frequently in pGBMs than sGBMs (*p* = 0.015). However, PLR did not differ between pGBMs and sGBMs (*p*=0.765), nor did LMR (*p* = 0.741, Table 2). No difference was found in NLR (*p* = 0.574), PLR (*p* = 0.966) or LMR (*p* = 0.564) with respect to IDH mutation status. We found no significant correlation between NLR or PLR and patients' age, gender, KPS, tumor location or size, or molecular markers. (Data not shown)

**Table 2:** Correlation of inflammation markers with molecular markers

Inflammation marker	Stratification	Histopathology		*P*	IDH-1R132H		*P*
		pGBM	sGBM		mutant	wild-type	
NLR	≤ 4.0	114	25	0.015	27	112	0.574
	> 4.0	27	0		4	23	
PLR	≤ 175.0	109	20	0.765	24	105	0.966
	> 175.0	32	5		7	30	
LMR	≤ 3.7	46	9	0.741	12	43	0.464
	> 3.7	95	16		19	92	

### Analysis of NLR, PLR, and LMR in predicting outcomes

**Figure 1:**
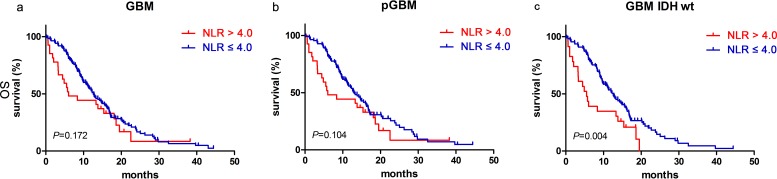
NLR predicted OS in glioblastomas Kaplan-Meier method with log rank test for NLR in predicting OS for **a**. glioblastomas, **b**. pGBM and **c**. IDH wt GBM.

We found that NLR had no significant prognostic value for patients with glioblastomas [12.80 months (95% CI 10.40–15.20) vs. 6.03 months (95% CI 1.16–10.90); p=0.172, Figure 1a] and those in the pGBM group [13.30 months (95% CI 1.91–15.69) vs. 6.03 months (95% CI 1.16–10.90); p=0.104, Figure 1b]. However, patients who had a NLR ≤ 4.0 and were in the group carrying IDH-1^R132H-wt^ had better prognoses [12.60 months (95% CI 10.22–14.98) vs. 5.50 months (95% CI 3.40–7.60); p=0.004, Figure 1c].

**Figure 2:**
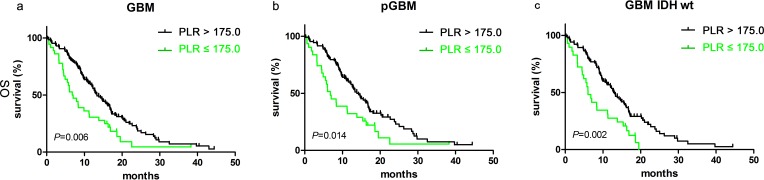
PLR predicted OS in glioblastomas Kaplan-Meier method with log rank test for PLR in predicting OS for **a**. glioblastomas, **b**. pGBM and **c**. IDH wt GBM.

The median OS of 13.33 months (95% CI 11.25–15.41) for patients with PLR ≤ 175.0 was longer than the 7.00 months (95% CI 4.22–9.78) for patients with PLR > 175.0 (p=0.006, Figure 2a). PLR ≤ 175.0 was also associated with better clinical outcome in the pGBM group [14.27 months (95% CI 11.83–16.71) vs. 6.80 months (95% CI 3.57–10.03); p=0.014, Figure 2b] and the IDH-1^R132H-wt^ group [13.00 months (95% CI 10.84–15.16) vs. 6.03 months (95% CI 3.38–7.68); p=0.002, Figure 2c].

**Figure 3:**
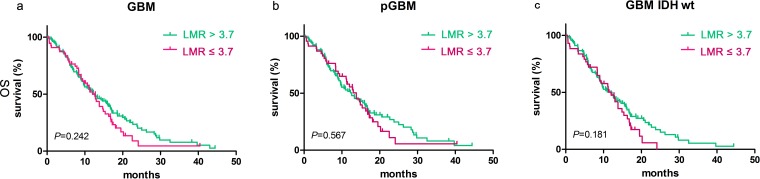
LMR didn't predict OS in glioblastomas Kaplan-Meier method with log rank test for LMR in predicting OS for **a**. glioblastomas, **b**. pGBM and **c**. IDH wt GBM.

The median OS did not differ significantly between groups stratified based on LMR ≥ 3.7 [12.00 months (95% CI 9.94–14.06) vs. 12.60 months (95% CI 9.19–16.00); p=0.242, Figure 3a]. No significant prognostic value for LMR ≥ 3.7 was observed in patients with pGBM [13.83 months (95% CI 10.74–16.92) vs. 12.60 months (95% CI 8.82–16.39); p=0.567, Figure 3b], nor with IDH-1^R132H-wt^ [12.00 months (95% CI 9.56–14.44) vs. 12.27 months (95% CI 9.07–15.47); p=0.181, Figure 3c].

Multivariate analysis indicated that age (p=0.022), extent of resection (p=0.034), full treatment (p=0.000) and IDH mutations (p=0.029) were independent prognostic factors after taking age, gender, KPS, extent of resection, full treatment, pathology and IDH mutations into account (Table 3). However, NLR, PLR, and LMR were strongly correlated with each other [(Spearman's rho coefficients of 0.631 (NLR vs PLR, p=0.000), −0.344 (NLR vs LMR, p=0.000) and -0.240 (PLR vs LMR, p=0.002)]. All three factors were analyzed in the multivariate analysis adjusted by the above 7 factors. Both NLR (HR=1.714, 95% CI 1.026-2.864, p=0.039) and PLR (HR=2.068, 95% CI 1.296-3.300, p=0.002) were indicated to be independent prognostic factors. However, we found that LMR had no independent prognostic value for OS (HR=0.733, 95% CI 0.481-1.119, p=0.150).

**Table 3:** Multivariate analysis of prognostic factors

Prognostic factors	HR	95% CI		*P* value
age	1.636	1.073	2.495	0.022
gender	0.988	0.664	1.471	0.953
Preoperative KPS	1.250	0.837	1.866	0.276
Pathology	1.362	0.687	2.701	0.376
Resection	1.531	1.033	2.268	0.034
Standard treatment	2.445	1.573	3.802	0.000
IDH-1R132H mutation	1.993	1.074	3.698	0.029

## DISCUSSION

In the present study, we first assessed the prognostic value of NLR, PLR, and LMR in glioblastomas, taking into account IDH mutation status. NLR and PLR were independent prognostic biomarkers for patient outcomes and therefore confirm published data from glioblastomas [21–23] and other malignancies [14, 17, 24]. However, LMR was not predictive of OS in glioblastomas.

We found that reduced NLR was associated with improved OS in pGBM, though the significance was not as obvious as in previous studies [21–23]. This difference may be explained by differences among previous studies. While NLR was established as a prognostic marker for malignancies in some studies [14, 17], others failed to observe a significant prognostic value for NLR in breast cancer [25], gastric cancer [26], and prostate cancer [27]. It is likely that not all patients received the same treatment in each study. In our study, all patients underwent surgery. Among them, 61.44% (102/166) had a gross treatment resection, and 68.67% (114/166) received radiochemotherapy according to Stupp's protocol. In other studies, the proportion of patients choosing each treatment strategy, which included biopsy, surgery, and radiochemotherapy, varied [21–23]. Additionally, the inclusion of IDH mutations was superior to histopathology alone for classifying glioblastomas [28]. We concluded that IDH-wt glioblastomas had better defined clinical outcomes than pGBM. Our multifactorial analysis first took IDH mutations as prognostic indicators, and NLR remained an independent prognostic biomarker. Interestingly, we observed that higher NLRs were more frequent in pGBM than sGBM. Zadora et al. reported that NLR values differed among glioma grades and were highest in glioblastomas [20]. Secondary glioblastoma originates from a lower-grade glioma. This likely explains why NLRs were low in sGBM. Furthermore, we also found that elevated PLR correlated closely with poor prognosis in our study, which is consistent with Han's results [23]. The prognostic value of PLR was found not only in IDH-wt glioblastomas, but also in glioblastomas and pGBMs in our study.

The mechanism underlying the prognostic role of NLR/PLR remains unclear in glioblastomas. The blood-brain-barrier is frequently disrupted in glioblastomas, allowing circulating lymphocytes to cross [29]. Moreover, NLR was significantly related to high neutrophil and low CD3^+^ T-cell infiltration into glioblastomas [23]. Tumor-infiltrating lymphocytes (TILs), which are predominately regulatory T cells in the glioblastoma microenvironment, could suppress immune responses [30]. However, recent studies indicate that TILs are not sufficient to mediate the glioblastoma-related immune suppression [31–33]. PD-L1 (programmed death ligand 1) and CTLA-4 (Cytotoxic T-lymphocyte-associated protein 4) have been identified as alternatives for immunosuppression in glioblastomas [34, 35]. Additionally, PD-L1 proteins were detected in the microenvironment of glioblastomas or brain metastases [36–38]. These results suggest a more complicated immunosuppressive mechanism in glioblastomas, which is likely to involve both systemic and local microenvironmental inflammation. We therefore propose that a complete score system is needed to fully assess systemic inflammation status, involving an immunosuppressive biomarker in the microenvironment.

## MATERIALS AND METHODS

### Study population

This retrospective study was conducted to investigate the relationship between NLR/PLR and glioblastomas. The inclusion criteria were: (1) Surgical treatment in Sanbo Brain Hospital from 2009 to 2014, (2) the presence of histologically confirmed supratentorial glioblastomas, (3) operative blood test performed prior to corticosteroid treatment or no chemotherapy within the previous month, (4) available medical records indicating the patient's age, gender, molecular pathology and follow-up data, and (5) provided informed consent before the investigation. Ultimately participating in the study were 166 patients, including 141 with pGBMs and 25 with sGBMs. The Stupp protocol was used for concurrent chemoradiotherapy followed by consolidation chemotherapy with temozolomide [39]. OS time was defined as the interval from surgery until death or the latest follow-up. All experiments using human tissues were approved by the Institutional Review Board of Sanbo Brain Hospital.

### Immunohistochemistry

Immunohistochemistry was used for detection of IDH mutations. The procedures were performed as described previously [9] using primary antibodies against IDH1^R132H^ (Dianova 1:100) . The cutoff values were 10% for IDH-1^R132H mut^.

### Statistics

Data are presented as means ± SEM. SPSS 22.0 was used for all the other statistical analyses. The χ2 test was used to evaluate the correlations between NLR, PLR and LMR and the patients' clinical characteristics. Survival curves were analyzed using the Kaplan-Meier method and the Breslow test. Values of *p*<0.05 (two-sided) were considered statistically significant.
